# Agronomic Traits and Molecular Marker Identification of Wheat–*Aegilops caudata* Addition Lines

**DOI:** 10.3389/fpls.2017.01743

**Published:** 2017-10-12

**Authors:** Wenping Gong, Ran Han, Haosheng Li, Jianmin Song, Hongfei Yan, Genying Li, Aifeng Liu, Xinyou Cao, Jun Guo, Shengnan Zhai, Dungong Cheng, Zhendong Zhao, Cheng Liu, Jianjun Liu

**Affiliations:** ^1^Crop Research Institute, Shandong Academy of Agricultural Sciences/Key Laboratory of Wheat Biology and Genetic Improvement in the Northern Yellow-Huai Rivers Valley of Ministry of Agriculture/National Engineering Laboratory for Wheat and Maize, Jinan, China; ^2^College of Plant Protection, Agricultural University of Hebei, Baoding, China; ^3^College of Life Science, Shandong Normal University, Jinan, China

**Keywords:** *Aegilops caudata*, agronomic traits, disease resistance, molecular marker, chromosome rearrangement

## Abstract

*Aegilops caudata* is an important gene source for wheat breeding. Intensive evaluation of its utilization value is an essential first step prior to its application in breeding. In this research, the agronomical and quality traits of *Triticum aestivum*-*Ae. caudata* additions B–G (homoeologous groups not identified) were analyzed and evaluated. Disease resistance tests showed that chromosome D of *Ae. caudata* might possess leaf rust resistance, and chromosome E might carry stem rust and powdery mildew resistance genes. Investigations into agronomical traits suggested that the introduction of the *Ae. caudata* chromosome in addition line F could reduce plant height. Grain quality tests showed that the introduction of chromosomes E or F into wheat could increase its protein and wet gluten content. Therefore, wheat-*Ae. caudata* additions D–F are all potentially useful candidates for chromosome engineering activities to create useful wheat-alien chromosome introgressions. A total of 55 EST-based molecular markers were developed and then used to identify the chromosome homoeologous group of each of the *Ae. caudata* B–G chromosomes. Marker analysis indicated that the *Ae. caudata* chromosomes in addition lines B to G were structurally altered, therefore, a large population combined with intensive screening pressure should be taken into consideration when inducing and screening for wheat-*Ae. caudata* compensating translocations. Marker data also indicated that the *Ae. caudata* chromosomes in addition lines C–F were 5C, 6C, 7C, and 3C, respectively, while the homoeologous group of chromosomes B and G of *Ae. caudata* are as yet undetermined and need further research.

## Introduction

*Aegilops caudata* L. [syn. *Ae. markgrafii* (Greuter) Hammer] is an annual diploid species (2*n* = 2x = 14, genome CC), naturally occurring mainly in the Aegean Region and in western Turkey, less commonly and more sporadically in inland Turkey and through the Fertile Crescent (Kilian et al., 2011). *Ae. caudata* can form dense stands, often together with other *Aegilops* species. Collected annual rainfall data indicate a range of 300–700 mm. From sea level up to 1,850 m (Kilian et al., 2011). *Ae. caudata* has been found to be resistant to wheat stripe rust (*Puccinia striiformis* Westend) (Valkoun et al., 1985; Baldauf et al., 1992; Toor et al., 2016), leaf rust (*P. recondita* Roberge ex Desmaz. f. sp. *tritici*) (Gill et al., 1985; Valkoun et al., 1985; Iqbal et al., 2007; Riar et al., 2012), stem rust (*Puccinia graminis* f. sp. *tritici*) (Valkoun et al., 1985; Dyck et al., 1990), powdery mildew (*Blumeria graminis* f. sp. *tritici*) (Gill et al., 1985; Valkoun et al., 1985; Baldauf et al., 1992), barley yellow dwarf luteovirus (Makkouk et al., 1994), snow mold (*Typhula ishikariensis* S. Imai) (Iriki et al., 2001), greenbug [*Schizaphis graminum* (Rondani)] (Baldauf et al., 1992) and hessian fly [*Mayetiola destructor* (Say)] (Gill et al., 1985). Moreover, some species of *Ae. caudata* has freezing tolerance (Barashkova and Migushova, 1984; Iriki et al., 2001), salt tolerance (Gorham, 1990), and could be used for iron and zinc fortification (Wang et al., 2011). Therefore, *Ae. caudata* is an excellent gene source for wheat improvement.

Wheat-*Ae. caudata* amphiploids, addition, substitution and translocation lines are bridging materials for transferring desirable genes from *Ae. caudata* to wheat. The creation and identification of these bridge materials is the first step in the gene transfer procedure. Muramatsu (1973) produced and identified a bread wheat-*Ae. caudata* 5C (5A,5D) substitution. Biithner et al. (1988) created a set of bread wheat (cv. Alcedo)-*Ae. caudata* addition lines, temporarily named as additions A to G (Schubert and Bluthner, 1995). Later, Friebe et al. (1992) described the C-banded karyotype of this set of addition lines except addition A. Latter, Molnár et al. (2016) studied the homoeologous relationships of flow sorted wheat and *Ae. caudata* chromosomes using COS markers using this set of additions. More recently, Danilova et al. (2017) used single gene FISH and exome capture sequencing approaches and revised the nomenclature of *Ae. caudata* chromosomes A, B, C, D, E, F and G to 1C, 2C, 5C, 6C, 7C, 3C, and 4C, respectively. Moreover, Kong et al. (1999a) also synthesized a *Triticum durum*-*Ae. caudata* amphiploid, and identified chromosome translocations within the backcrossed progenies between wheat and the *T. durum*-*Ae. caudata* amphiploid. However, less useful markers for C chromosome were available in transferring genes from *Ae. caudata* into wheat.

Comprehensive evaluation of the disease resistance status, agronomical characters and quality traits of wheat-*Ae. caudata* addition lines will provide useful background information for future research to create useful wheat-*Ae. caudata* chromosome translocations for wheat breeding programs. Although the wheat-*Ae. caudata* addition lines A–G have been produced and identified (Schubert and Bluthner, 1995), the breeding value of this set of material has not yet been evaluated which stymied the creation and utilization of compensating wheat-*Ae. caudata* translocations. In this research, the level of disease resistance, agronomical characteristics and quality traits of wheat-*Ae. caudata* addition lines B–G (addition A was not available) were investigated or measured. Moreover, EST-based molecular markers specific for *Ae. caudata* chromosomes were developed to identify the homoeologous group of *Ae. caudata* chromosomes.

## Materials and methods

### Plant material

*Triticum aestivum* cv. Alcedo (ALCD), ALCD-*Ae. caudata* additions B–E (TA3558-TA3561), and G (TA3563) (Schubert and Bluthner, 1995) were provided by Prof. WX Liu, College of Life Science, Henan Agricultural University. *Ae. caudata* (TA1908), ALCD-*Ae. caudata* additions F (TA3562) (Schubert and Bluthner, 1995) and *T. turgidum* (TA10543) were provided by Prof. BS Gill, Wheat Genetic and Genomic Resource Center, Kansas State University. *T. aestivum* cv. Chinese Spring (CS), Mianyang11 (MY11) and Mianyang15 (MY15) were provided by Prof. ZJ Yang, School of Life Science and Technology, University of Electronic Science and Technology of China.

### Disease resistance testing

Stripe rust, leaf rust, stem rust and powdery mildew resistances of 20 individual plants of each of ALCD-*Ae. caudata* additions B–G, ALCD, TA1908, CS, MY11, and MY15 were tested. Among these lines, CS, MY11, and MY15 are highly susceptible to all four diseases, hence the disease response scoring did not begin until these three control genotypes were fully infected. The disease responses were scored on a 0–4 rating scale according to Wang et al. (2014), whereas 0 indicates immune, 0; means nearly immune but showing a small fleck on the leaf, 1 indicates highly resistant, 2 means moderately resistant, 3 indicates moderately susceptible, and 4 means highly susceptible. Record disease resistance levels (DRL) of 20 individual plants of each material truthfully, if there are resistance segregations, for example, the DRL of some plants are 1, some are 3, record as 1, 3. If the DRL of all 20 individual plants are completely same, just record only one DRL value.

The pathogen inoculation methods of stripe rust, leaf rust and powdery mildew were according to Liu et al. (2013), while stem rust inoculation was according to Wu et al. (2014). Stripe rust resistance was determined on both seedlings and adult plants using isolates of races CY32, CY33, and Su-4 in the experimental farmland of School of Life Science and Technology, University of Electronic Science and Technology of China. Stem rust resistance was determined on seedlings using mixed isolates of 34MKGQM and 21C3CTHSM in the greenhouse of College of Plant Protection, Shenyang Agricultural University. Leaf rust resistance was determined on seedlings using mixed leaf rust isolates of THTT, PHTT, THKS, THTS, and THKT in the greenhouse of College of Plant Protection, Agricultural University of Hebei. Powdery mildew resistance was determined on both seedlings (in greenhouse) and adult plants (field) following inoculation with mixed powdery mildew races collected from four different cities including Jinan, Linyi, Dezhou and Heze of Shandong Province.

### Agronomical trait investigation and quality measurement

ALCD and ALCD-*Ae. caudata* addition lines B–G were planted in the farmland at four different cities including Jinan, Dezhou, Heze and Linyi of Shandong Province on October 25, 2015. The experimental design consisted of three biological replications arranged in a randomized block, and all plots and sites followed the same standard cultivation practices and were grown under irrigated conditions. Spacing between plants in a row was 20 cm and the between row spacing was 33 cm. Each experimental plot contains seven rows. A border buffer consisting of 18 rows of wheat variety Jimai22 surrounded the experimental plots so as to eliminate the margin effect. The four cities were in a temperate continental monsoon climate, characterized by dry, cold winters and rainy, hot summers. During wheat growing season, total precipitation in 2016 was 264.7 mm in Jinan, 176.3 mm in Dezhou, 279.4 mm in Heze, and 339.6 mm in Linyi, respectively. Averaged temperatures in 2016 growing season were 11.5°C in Jinan, 9.7°C in Dezhou, 10.5°C in Heze and 11.0°C in Linyi, respectively. Soil types of all four stations were fluvo-aquic soil, same amount of compound fertilizer was used, weeds and diseases were controlled. The climate information was obtained from the official website of Shandong Meteorological Bureau. The soil type information of four cities was obtained from the official website of Soil and Fertilizer Station, Shandong Provincial Department of Agriculture.

Randomly select 10 plants of each material for the measurement of plant height, spike length (the selected spikes were painted by red lacquer using a manual spray painting pot), flag leaf length and width, tiller number, spikelet number before leaf rolling or shrinking on May, 2016. Randomly harvest one spike of each individual plants (the 10 spikes painted by red lacquer were included) after they were fully mature in June, 2016. Spikes were threshed manually to prevent seed loss so as to determine grain number of the 30 spikes and thousand-kernel weight. Data on the number of tillers, grain number of 30 spikes and thousand-kernel weight from Jinan was not obtainable.

Grain samples for quality tests were collected from four cities of Shandong Province as mentioned above. The grains were milled using wheat grinding machine 3100 (Perten, Sweden), the protein content was measured with a near-infrared (NIR) spectrometer DA7200 (Perten, Sweden) according to the approved method 46-12 (AACC, 2000), three replications for each sites. The wet gluten content was measured with a gluten tester 2200 (Perten, Sweden), three replications for each sites. Data processing and *t*-test was performed by Microsoft Excel 2010 and the statistical software SPSS v. 13.0. The data from four sites were completely consistent with each other (tiller number, grain number of 30 spikes and thousand-kernel weight, across the three cities), the trait variation when compared to the background genotype ALCD will be regarded as attributable to the presence of the alien chromatin. Alternatively, it might be considered as a result of interaction of genotype and environments. In this research, only the former will be discussed.

### DNA isolation, primer design and PCR

Total genomic DNA was prepared from young leaves using the SDS protocol (Liu et al., 2006). A total of 410 bin mapped Expressed Sequence Tags (ESTs) were selected from the wheat EST mapping project (http://wheat.pw.usda.gov/NSF/data.html) for EST-Sequence Tagged Site (EST-STS) primer design using the software Primer 3 (http://frodo.wi.mit.edu). EST-STS PCR amplifications were performed as described by Gong et al. (2014). To obtain higher levels of polymorphism, the PCR products were digested with the 4-base cutter enzymes *Alu*I, *Hae*III, *Msp*I or *Rsa*I. The PCR products were separated on a 2% agarose gel.

A total of 258, 107 and 185 EST-Simple Sequence Repeat (EST-SSR), Conserved Orthologous Sequence (COS) and PCR-based Landmark Unique Gene (PLUG) primers were selected and synthesized, and PCR protocol were followed that according to Xue et al. (2008), Quraishi et al. (2009), and Ishikawa et al. (2007), respectively. To obtain high levels of polymorphism, the PLUG PCR products were digested with the four-base cutter enzymes *Hae*III or *Taq*I according to Ishikawa et al. (2007), whereas the COS and EST-SSR PCR products were separated on a native polyacrylamide gel electrophoresis and stained in a silver solution according to Xue et al. (2008) and Quraishi et al. (2009). The molecular markers specific for *Ae. caudata* chomosomes were determined using *Ae. caudata* accession TA1908, ALCD-*Ae. caudata* addition lines TA3598-TA3563 as positive control and wheat genotypes Alcedo, CS, *T. turgidum* accession TA10543, MY11 and MY15 as negative control.

## Results

### Disease resistance tests of ALCD-*Ae. caudata* additions

In this research, wheat stripe rust, leaf rust, stem rust and powdery mildew resistance of *Ae. caudata*, the ALCD- *Ae. caudata* B–G addition lines, and wheat controls ALCD, CS, MY11, and MY15 were tested (Table 1). The results showed that CS, MY11 and MY15 were highly susceptible to all four diseases, indicating that the infection races were fully inoculated. *Ae. caudata*, ALCD and ALCD-*Ae. caudata* B–G additions were nearly immune or highly resistant to stripe rust at the seedling and adult plant stages, suggesting that there is at least one stripe rust resistant gene in the six additions which was derived from the wheat line ALCD. *Ae. caudata* and the ALCD-*Ae. caudata* D addition line were highly resistant to leaf rust, while ALCD and other five additions were susceptible, indicating that D chromosome of *Ae. caudata* might carry a leaf rust resistance gene. *Ae. caudata* and the ALCD-*Ae. caudata* E addition line were highly resistant to stem rust and powdery mildew, while ALCD and other five additions were susceptible, indicating that E chromosome of *Ae. caudata* might possess stem rust and powdery mildew resistant gene (s).

**Table 1:** Stripe rust, leaf rust, stem rust and powdery mildew resistances of ALCD-*Ae. caudata* additions.

**Accession No**.	**Material**	**Infection with stripe rust**	**Infection with leaf rust**	**Infection with stem rust**	**Infection with powdery mildew**
TA1908	*Ae. caudata*	0;	0;	0;	0
ALCD	Alcedo (*Triticum aestivum*)	0;	3	3	4
TA3558	ALCD-*Ae. caudata* addition B	0; 1	3	3	4
TA3559	ALCD-*Ae. caudata* addition C	0; 1	4	3	3
TA3560	ALCD-*Ae. caudata* addition D	1	0; 1	3	3
TA3561	ALCD-*Ae. caudata* addition E	1	3	1	0
TA3562	ALCD-*Ae. caudata* addition F	1	3	4	4
TA3563	ALCD-*Ae. caudata* addition G	1	4	4	4
CS	Chinese Spring	4	4	4	4
MY11	Wheat variety Mianyang11	4	4	4	4
MY15	Wheat variety Mianyang15	4	4	4	4

*All the four wheat diseases listed in this table are scored using a 0–4 scale, whereas 0 indicates immune,; means nearly immune, 1 indicates highly resistant, 3 indicates moderately susceptible, and 4 means highly susceptible. 0; 1 means that the resistance level of some plants was nearly immune, and some are highly resistant. The stipe rust and powdery mildew resistance levels of material tested at the seedling and adult plant stages are completely same, therefore, only one stipe rust and powdery mildew resistance level of each material were listed herein*.

### Agronomic trait investigation

**Figure 1:**
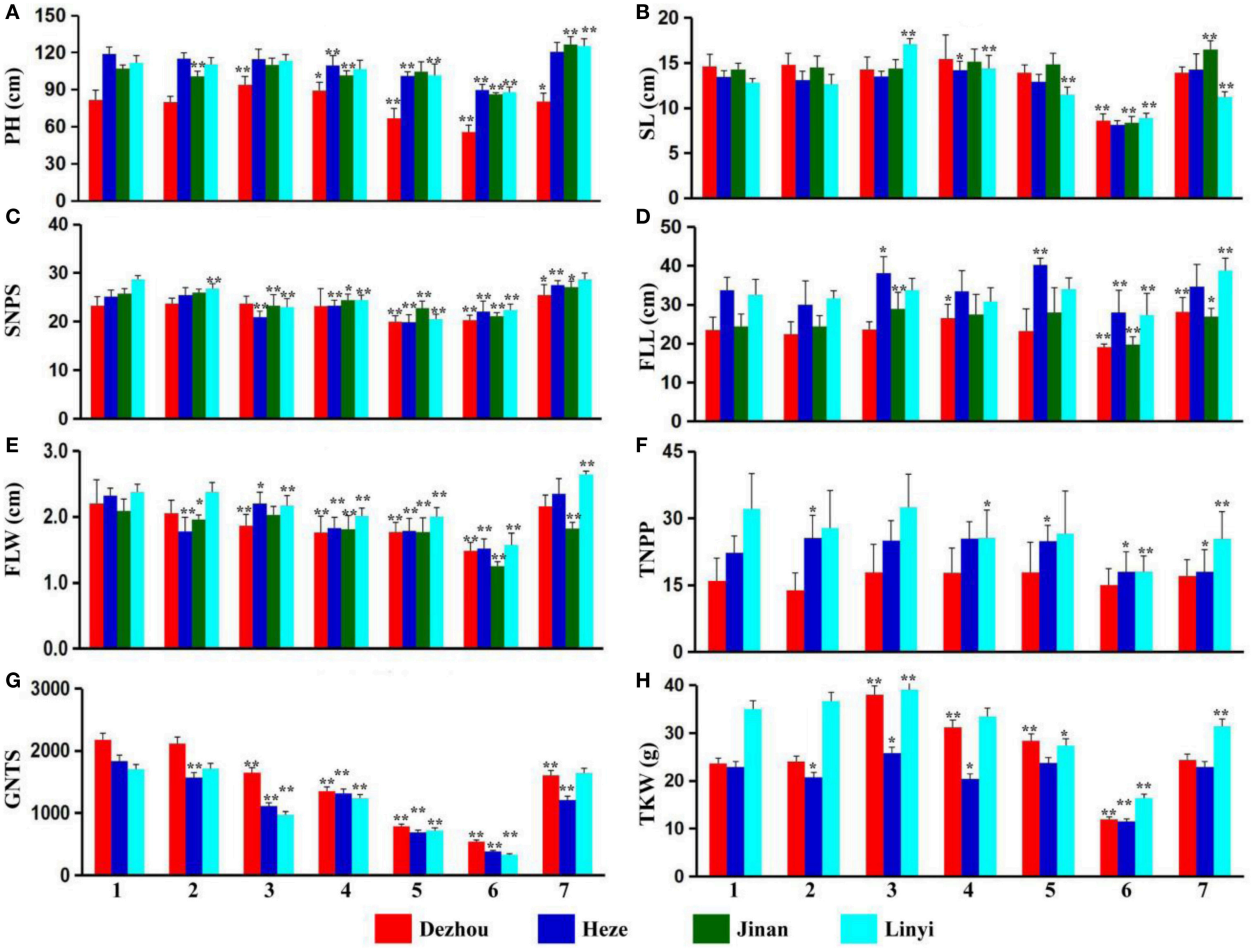
Agronomical traits investigation result of the material tested. Spikelet number, grain number per 30 spikes and thousand-kernel weight data of Ji'nan are not obtained due to crop rotation.1–7 represent *T. aestivum* cv. Alcedo, Alcedo-*Ae. caudata* B to G addition lines. PH **(A)**, SL **(B)**, SNPS **(C)**, FLL **(D)**, FLW **(E)**, TNPP **(F)**, GNTS **(G)**, and TKW **(H)** are the abbreviations of Plant Height, Spike Length, Spikelet Number Per Spike, Flag Leaf Length, Flag Leaf Width, Tiller Number Per Plant, Grain Number of 30 Spikes and Thousand Kernel Weight, respectively. ^*^significant at *P* < 0.05 by *t*-test as compared to relative data of ALCD; ^**^significant at *P* < 0.01 by *t*-test as compared to relative data of ALCD. Bar represents standard deviation.

Plant height, spike length, spikelet number and five other agronomic traits of ALCD and ALCD-*Ae. caudata* B–G additions were studied. The results showed that there were no significant agronomic trait influences with the addition of chromosome B of *Ae. caudata* into ALCD (Figures 1A–H). Chromosome C of *Ae. caudata* introduced into ALCD showed an increase in the thousand-kernel weight (Figure 1H), however, that chromosome also seemed to produce a negative impact on grain number per spike (Figure 1G). Wheat plants carrying chromosome D of *Ae. caudata* showed reduced flag leaf width and decreased grain number per spike (Figures 1E,G) compared with ALCD. Chromosome E of *Ae. caudata* reduced wheat flag leaf width (Figure 1E), and had a negative influence on spikelet number and grain number per spike (Figures 1C,G). Chromosome F appeared to reduce plant height (Figure 1A), but had a negative influence on nearly all of the other agronomic traits (Figures 1B–H). The introduction of chromosome G into wheat had no significant influence on wheat agronomical traits due to the fact that data from four or three locations were not consistent with each other (Figure 1G).

### Quality trait measurements

**Figure 2:**
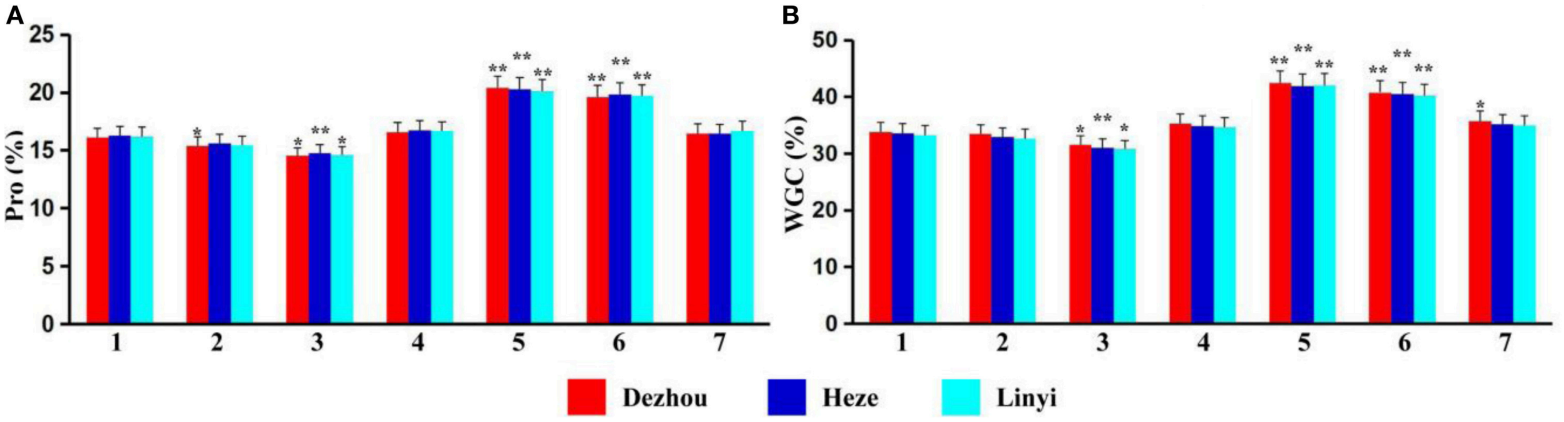
Protein and wet gluten contents of the material tested. Pro **(A)**, Protein; WGC **(B)**, Wet Gluten Content; 1–7 represent *T. aestivum* cv. Alcedo, Alcedo-*Ae. caudata* B to G addition lines. ^*^significant at *P* < 0.05 by *t*-test as compared to relative data of ALCD; ^**^significant at *P* < 0.01 by *t*-test as compared to relative data of ALCD. Bar represents standard deviation.

Protein content and wet gluten content of ALCD and ALCD-*Ae. caudata* B–G addition lines were measured, and the results showed that data from Dezhou, Heze and Linyi were similar across all sites (Figures 2A,B). Protein content and wet gluten content of ALCD were 16.1–16.3% (Figure 2A) and 33.3–33.8% (Figure 2B), respectively, while measurements of 15.4–20.4% (Figure 2A) (protein content) and 30.8–42.5% (Figure 2B) (wet gluten content) were recorded for the for B–G additions. There were no significant quality differences with the introduction of chromosomes B, D, and G of *Ae. caudata* into ALCD (Figures 2A,B). However, the presence of chromosome C significantly reduced both wheat protein and wet gluten contents, while chromosomes E and F significantly increased wheat protein and wet gluten contents.

### Identification of ALCD-*Ae. caudata* additions using molecular markers

**Figure 3:**
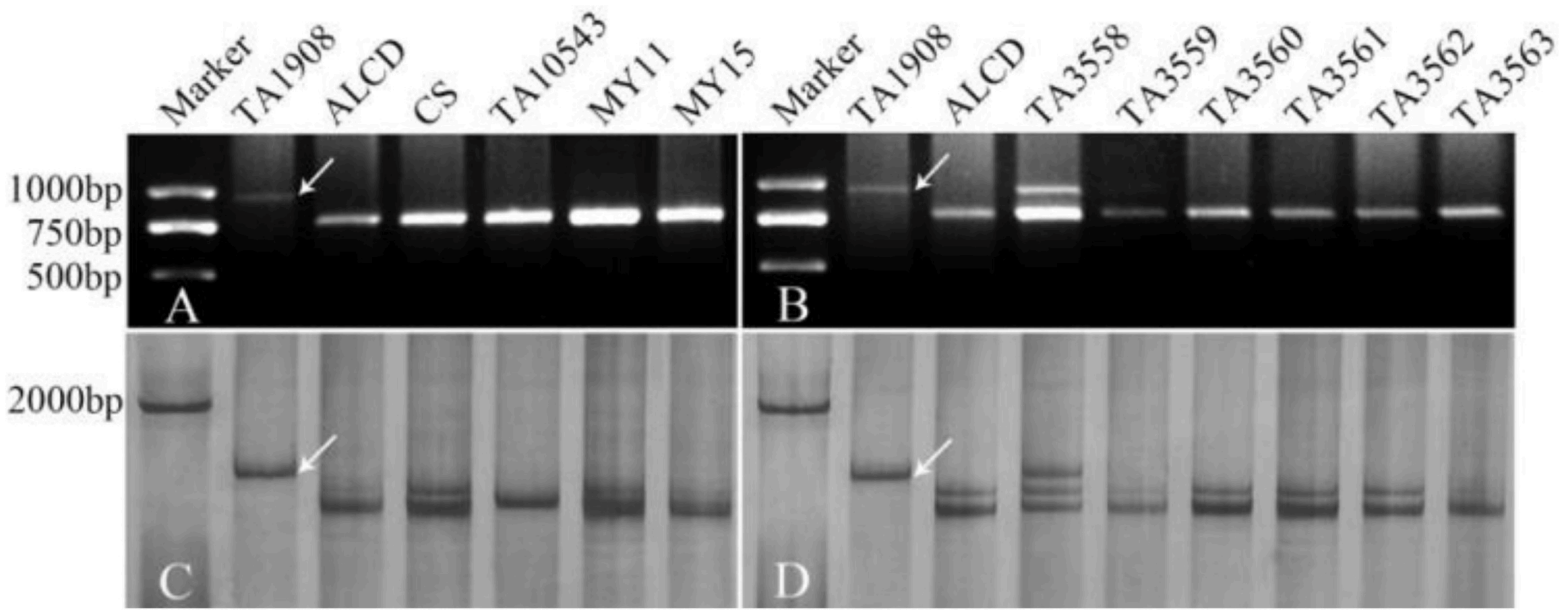
PCR patterns of primer TNAC1497 **(A,B)** and TNAC1605 **(C,D)**. Arrows indicate polymorphic bands; Panels **A,B** are from agarose gel, while **C,D** are from polyacrylamide gel. TA1908 represents *Ae. caudata*, while ALCD, CS, MY11, and MY15 mean *T. aestivum* cv. Alcedo, cv. Chinese Spring, cv. Mianyang11 and cv. Miyang15, respectively. TA10543 means *T. turgidum*, TA3598-TA3563 mean ALCD-*Ae. caudata* additions B–G, respectively.

*Ae. caudata*, CS, *T. turgidum*, MY11 and MY15 were used to screen 410 pairs of EST-STS primers, 258 pairs of EST-SSR primers, 107 pairs of COS primers and 185 pairs of PLUG primers. The results showed that 77 of EST-STS primer pairs (18.7% of the total primer pairs tested), 46 of EST-SSR primer pairs (17.8%), 21 of the COS primer pairs (19.6%) and 64 of the PLUG primer pairs (34.6%) could generate additional DNA band(s) from *Ae. caudata* compared to wheat controls as listed in Table 2. The PCR patterns of primer pairs TNAC1497 and TNAC1605 are shown in Figures 3A,C.

**Table 2:** Primers screened and relative information of molecular markers obtained.

**Primer**	**Number of primer screened**	Number of polymorphic primers^*^	**% polymorphism**	**Number of markers located on the addition**	**% markers located on the addition**
EST-STS	410	77	18.7	15	3.6
EST-SSR	258	46	17.8	13	5.0
COS	107	21	19.6	4	3.7
PLUG	185	64	34.6	23	12.4

* *Indicate additional DNA bands were amplified by comparing to wheat controls*.

PCR using the polymorphic primer pairs was performed on ALCD and ALCD-*Ae. caudata* B–G addition lines to locate the polymorphic bands to the *Ae. caudata* chromosomes. The results suggested that there were 15 (3.6% of the polymorphic primer pairs number), 13 (5.0%), 4 (3.7%), and 23 (12.4%) EST-STS, EST-SSR, COS, and PLUG polymorphic markers, respectively, that could be located to *Ae. caudata* chromosomes. The PCR patterns of primer pairs TNAC1497 and TNAC1605 as applied to DNA of the addition lines are shown in Figures 3B,D. The detailed information concerning the 55 markers developed by the current research is listed in Table 3.

**Table 3:** Markers specific for *Ae. caudata* chromosomes developed by the current study.

**No**.	**Primer**	**Primer type**	**Primer sequence (5′–3′)**	**Chromosome location**		**Enzyme used**	**Marker size (bp)**
				***T. aestivum***	***Ae. caudata***		
1	BF291891^#^	EST-STS	F:CATGGACATCGACAAGATCG R: GAGCTCCGTCGATATGAAGC	1DS5-0.70-1.00	B	–	750
2	MAG2282^*^	EST-SSR	F: ATGCCACTGGGGAGACAGTATG R: TGTAAGAACGTGGGATGATGCTG	1DS	B	–	350
3	BE446243^#^	EST-STS	F:CAAGGAGTGCAAGAAGCACA R:GTCGCCTCTTGCTTAAATGC	C-2DS1-0.33	B	–	830
4	BCD348^*^	EST-SSR	F: TTCACCGCCAAACACAGAGC R: CCCCTACCAAAGACTCCAAACG	2AS	B	–	400
5	BE499186^#^	EST-STS	F: CTGCTGCTCCTCCTGCTC R: ACCCCCATGGTCACTGTAAA	3DL3-0.81-1.00	B	–	600
6	MAG1242^*^	EST-SSR	F: GCCACCGACTGTTAGGTTTCACTC R: CGAGGGTTCTTGGGAATGACAC	5B	B	–	400
7	BE606912^#^	EST-SSR	F:CTGCAAAGACACCCAACAGA R:TCATCATGCACCATCAGTCA	2BS3-0.84-1.00	C	–	850
8	TNAC1497	TANC	F:ATCAAACCTGACGGTGTTCAG R:CATGCAGACTACAGGTCCAGA	5AS1-0.40-0.75 5BS4-0.43-0.56 5DS4-0.22-0.63	C	–	900
9	TNAC1605^*^	TNAC	F:TTGCCCTTGTTGTGAAGAATC R:TGTGCCATAGGCTCTCTTTGT	5AL12-0.35-0.57 5BL8-0.52-0.75 5DL1-0.60-0.69	C	–	1,500
10	TNAC1559	TNAC	F:AAACAAGGCCCTGAAACACTT R:CATTGTCAGGCTATGGGACAT	5AL10-0.57-0.78 5BL9-0.76-0.79 5DL5-0.76-1.00	C	*Taq*I	400
11	MAG1426^*^	EST-SSR	F: GCGAGTTTTCGTAGCAAAGG R: TCACAGGAGTGGAGGCTCAC	5B	C	–	300
12	BE494952^#^	EST-STS	F: GGAAGGATCCGACAACAAAA R: TTCTCCTCATCCCAATCGAC	5BS6-0.81-1.00	C, D	*Msp*I	500
13	CDO457^*^	EST-SSR	F: CCTTCTTTTCGCAGCCATATCG R: GTGGTCACGAGTGTCGGTACAAC	5AL	C, D	–	350
14	TNAC1002	TNAC	F:ATGTTGGAAGGATTGTCATCG R:ATCCTTAAAGGTGCGGCCATA	unknown	D	–	250
15	BE586140^#^	EST-STS	F: GATCCTCGTGATGCTGATGA R: GCCCAATGACCATCAATACC	1DS5-0.70-1.00	D	*Hae*III	350
16	TNAC1178	TNAC	F:TGATACCGAGGCTATCCACAT R:ACATGAACAAGGATCATGCTG	C-2AS-0.78 2BS11-0.27-0.53 2DS1-0.33-0.41	D	*Hae*III	400
17	TNAC1204	TNAC	F:GAGAGGAATGCGTGAAGTTTG R:AGACCATCTTTCCGGTCTTTG	2AL4-0.27-0.77 2BL7-0.50-0.58 2DL10-0.49-0.58	D	*Hae*III	260
18	BF293305^#^	EST-STS	F: GGCAATCATTATGGATGCTG R: GCGTTGCGTGACATCACTAT	5BS6-0.81-1.00	D	–	260
19	CDO1326^*^	EST-SSR	F:CCGTAACAAGCAACATAAAGGGTC R: TCACATCAGTCGTCTCTCGTCG	5AL	D	–	280
20	COS96^*^	COS	F:TGAGAAGCTTGAGGAGTTGG R:TCTCATGCAAACTATCTGCG	5AS1-0.40-0.75 5BS4-0.43-0.56	D	–	520
21	TNAC1688	TNAC	F:TGAAGTGTCAGTGCCCTTCTT R:GTCAAATCCAAGTTCCACGAG	6D	D	–	770
22	TNAC1719	TNAC	F:TCATAGCACATGCAGCAACA R:CGAGCTCGTTAGCTTCTCTGA	6B	D	–	500
23	TNAC1722	TNAC	F:CCAAGGTTATGATCCTTTCCA R:CCTGCTTCTGCACTGAAGTTT	6B	D	–	480
24	TNAC1735	TNAC	F:CGAATCTGTCAGGTGCAACA R:TGTCATGGAGTGTTTGCTGTC	6B 6D	D	–	1,400
25	TNAC1739	TNAC	F:ACATCGAGAAGATCGAGTTGC R:TGGAAGCCCAGTTCTCCTTAT	6B 6D	D	–	1,000
26	TNAC1728	TNAC	F:AAGGCGCTCACCCTTCTC R:GACGCTTCGGCTCGTCAC	6B 6D	D	*Hae*III	1,100
27	TNAC1673	TNAC	F:TCAGGTGGTACGTCGTCTTGT R:TTTGGAGTGATCGGAGCTG	6D	D	*Taq*I	320
28	TNAC1679	TNAC	F:TATTGGCTCAACCAACCATTC R:TTCCAAACCACCCAGTGTGTA	6AS5-0.65-1.00 6BS-Sat 6DS4-0.79-0.99	D	*Taq*I	900
29	TNAC1721	TNAC	F:TCCTGTTCTCGTTCCTAGGTG R:ATTGCAGAATCCATCCAATGA	6B	D	*Taq*I	250
30	TNAC1731	TNAC	F:TGTTGCTTTCAGAGCGAATTT R:TTGCTCCACCGAGATCACTAC	6B	D	*Taq*I	1,400
31	BE500714^#^	EST-STS	F: GTGTCTGTTGGACCTGCAAA R: GCAAGTGCACACAGGAGAAA	C-1DS3-0.48	E	–	710
32	BE637610^#^	EST-STS	F: TAGCACCCAAGGGAAGAAGA R: AGAGGATGTACCACGCCAGT	C-1DS3-0.48	E	*Msp*I	450
33	COS41^*^	COS	F:AAGGGGTTCATGGATAAAGG R:ACAGACAGAGCTTGTGAGCG	2AL1-0.85-1.00 2BL6-0.89-1.00 2DL9-0.76-1.00	E	–	400
34	TNAC1812	TNAC	F:ACTTCGCTTGGTCTCCTCAAT R:GAGAAGTGTGCCAATTCCAAA	7AL5-0.63-0.71 7BL7-0.63-0.78 7DL5-0.30-0.61	E	*Hae*III	920
35	TNAC1782	TNAC	F:TCACTGAACAGCCTAGACATGG R:ATTCGCAGACCGCATCTATC	7AS2-0.73-0.83 7BS2-0.27-1.00 7DS4-0.73-1.00	E	*Taq*I	400
36	MAG3047^*^	EST-SSR	F: CCACGCCAACAAGAGATTTT R: ACTGTGCCATGCTTACCAAT	7BL	E	–	400
37	TNAC1140	TNAC	F:TCCCAGAAATTACAAGGCTCA R:AGGAACCCTATGCATTGGAAA	2AL3-0.77-1.00 2BL6-0.89-1.00 2DL6-0.94-1.00	F	*Hae*III	600
38	COS38^*^	COS	F:ATCAACAAGATCTTCGACGG R:CTTTGTCTGAACATTGCTGC	2BL4-0.50-0.89 2DL9-0.76-1.00 C-2AL1-0.85	F	–	350
39	TNAC1296	TNAC	F:GCATCCTGTCCCTCATCAC R:TCGAGGTCTCTAGACCAATGC	3AS4-0.45-1.00 3BS9-0.57-0.78 3DS4-0.59-1.00	F	–	2,200
40	TNAC1359	TNAC	F:GTAAATAGCGCCATCTGCGTA R:CTCTGGATGCAGTTGGAATGT	3AL3-0.42-0.61 3BL3-0.41-0.50 3DL1-0.23-0.81	F	–	1,200
41	TNAC1367	TNAC	F:CCTCAACATCTCCAAGGATCA R:CCGCTGGATCTGATTAGGC	3AL5-0.78-0.85 3BL7-0.63-0.81 3DL1-0.23-0.81	F	–	950
42	BE442801^#^	EST-STS	F: CCTTTATGCAGCGAGTGTGA R: ATGCCATCCCATAGAACGAG	3BS8-0.78-1.00	F	*Hae*III	320
43	TNAC1294	TNAC	F:CGGAAACTTTAGCCTTCTGCT R:GTCGTGTCAGATGCTTTGGAT	3AS4-0.45-1.00 3BS9-0.57-0.78 3DS4-0.59-1.00	F	*Hae*III	750
44	MAG620^*^	EST-SSR	F: TAGTTGCATGGTCGCTTCTG R: CGTAGCTTTTCGTTGATCCC	3A	F	–	220
45	MAG905^*^	EST-SSR	F: ATGTGAATGGAAGGTCGGAG R: AGCACTTGCAGGCTCTTCAT	3AL	F	–	350
46	MAG501^*^	EST-SSR	F: CAGCACCAACATCAGATTGC R: CAGGCTTCATCCAAGAGAGG	3DS	F	–	220
47	MAG500^*^	EST-SSR	F: CAGCACCAACATCAGATTGC R: TCATGTACGGCTTCATCCAA	3DS	F	–	270
48	BE637804^#^	EST-STS	F:CGCAGTTGCAGAAATTGGTA R:GCAGTCCATTTGTTGGTTCC	1BL3-0.85-1.00	G	–	350
49	BE426818^#^	EST-STS	F:ATGGGGATTCCAAGATAGGG R:CGTTAGGCCTTTTGGGTACA	2BL6-0.89-1.00	G	*Msp*I	750
50	COS47^*^	COS	F:TGACGAAGAAGATCGAAAGG R:AAGAATGTTCAGCAACAGCC	2AL1-0.85-1.00 2BL6-0.89-1.00 2DL9-0.76-1.00	G	–	800
51	BE406551^#^	EST-STS	F: TGCTTCCGCAACTACATCAG R: TGGTGACCCACAACAGAATG	3DL3-0.81-1.00	G	–	520
52	BE403428^#^	EST-STS	F: ACTGTGATCCCCGACAGGTA R: GCAGGCCAAAACTGAATGTT	3DL3-0.81-1.00	G	*Hae*III	150; 250
53	MAG4194^*^	EST-SSR	F: CATCCACATCCAACAGCAAC R: CAACCCCAAGTCAGCATTTT	3AL	G	–	400
54	BE445831^#^	EST-STS	F: GTGCTTCAACTTCCCAAAGC R: CCCACAATGCTGTGTTTGTC	4BS1-0.81-1.00	G	–	680
55	MAG1682^*^	EST-SSR	F: CGAATGCCAAGCTGTTCCCT R: ACATGCCCCTTGAGAGTGTGG	4BL	G	–	260

* *PCR product separated on a native polyacrylamide gel. –,no restriction enzyme used*.

# *the primer pairs were newly developed*.

**Figure 4:**
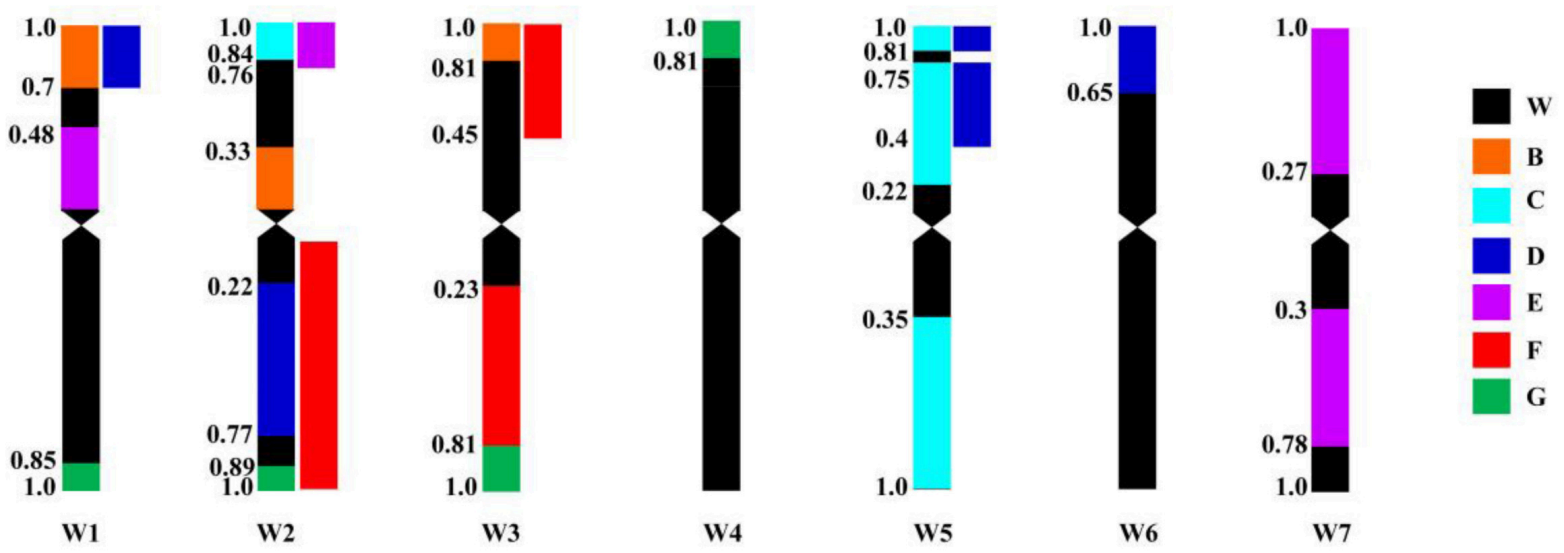
Schematic patterns of *Ae. caudata* chromosome rearrangement compared to wheat chromosomes as a reference revealed by PLUG markers physically mapped. *Ae. caudata* chromosomes were marked as B, C, D, E, F and G. W means wheat, W1–W7 represents wheat chromosome group 1–7, respectively. Define the lengths from centromere to both chromosome ends are 1, the values shown on the left of the chromosomes are fragment length (FL).

Primer pairs belong to homoeologous groups 1, 2, 3, and 5 could amplify polymorphic bands from ALCD-*Ae. caudata* B addition compared to wheat controls (Table 3; Figure 4), implying that a complicated rearrangement involving 1C, 2C, 3C, and 5C might have occurred to chromosome B of *Ae. caudata*. Primer pairs belong to homoeologous groups 2 and 5 could amplify polymorphic bands from ALCD-*Ae. caudata* C addition compared to wheat controls (Table 3; Figure 4), implying that a rearrangement involving 2C and 5C might have occurred to chromosome C of *Ae. caudata*. Molecular marker data physically mapped in wheat also showed that a rearrangement involving 2C, 5C, and 6C might have occurred to chromosome D of *Ae. caudata* (Table 3; Figure 4). Similarly, a 1C, 2C, and 7C rearrangement might have occurred to chromosome E of *Ae. caudata* (Table 3; Figure 4), a 2C and 3C rearrangement might have occurred to chromosome F of *Ae. caudata* (Table 3; Figure 4), a 1C, 2C, 3C, and 4C rearrangement might have occurred to chromosome G of *Ae. caudata* (Table 3; Figure 4).

## Discussion

The C genome of *Ae. caudata* is known to carry many useful genes that can be used for wheat breeding. Whilst investigating the potential of exploiting useful genes from the C genome of *Ae. caudata*, Riar et al. (2012) mapped a leaf rust resistance gene *LrAC* originating from a wheat-*Ae. caudata* introgression line onto the short arm of chromosome 5D of wheat. The *LrAc* gene is a homoeoallele of an ortholog *Lr57*. Iqbal et al. (2007) mapped an *Ae. caudata*-derived major leaf rust resistant quantitative trait locus (*Qlr.ipk-2A*) on chromosome 2AS of wheat. Weidner et al. (2012) mapped two *Ae. caudata*-derived powdery mildew resistance loci, *QPm.ipk-1A* and *QPm.ipk-7A*, on wheat chromosome arms 1AS and 7AL, respectively. Toor et al. (2016) mapped an *Ae. caudata*-derived stripe rust resistance gene on wheat arm 5DS. Dyck et al. (1990) selected stem rust resistant germplasm from cross combinations of *Ae. caudata* and the 5B monosomics of wheat. None of the afore-mentioned studies indicated which specific chromosome of *Ae. caudata* was carrying the resistance gene(s). The C genome of *Ae. caudata* has been found to be the source of the C genome of *Ae. triuncialis* L. (2*n* = 4x = 28, CCUU) and *Ae. cylindrica* Host (2*n* = 4x = 28,CCDD) (Kong et al., 1999a,b). However, there have been earlier reports of disease or pest resistance found in these latter species which clearly has originated from the U or D genomes (not the C genome) (Martin-Sanchez et al., 2003), or alternatively, the resistance was derived from the C genome but the specific identity of that C chromosome based on its conformity to a Triticeae homoeologous group remained unknown (Romero et al., 1998; Galaev et al., 2006; Kuraparthy et al., 2007; Ghazvini et al., 2012). In this current research, we found that the D chromosome of *Ae. caudata* might possess a leaf rust resistant gene, and the E chromosome might possess stem rust and powdery mildew resistant gene (s). Therefore, these two C-genome chromosome addition lines deserve further investigations involving chromosome engineering activities to produce agronomically useful translocations.

Apart from evaluation of wheat-*Ae. caudata* germplasm for disease and pest resistance (Romero et al., 1998; Galaev et al., 2006; Kuraparthy et al., 2007; Ghazvini et al., 2012), reports of the agronomical and quality traits are rather rare. In this present research, both agronomical and quality traits of wheat-*Ae. caudata* additions B–G were investigated or measured, and the introduced *Ae. caudata* chromosomes into wheat appeared to bring negative influences to several agronomical traits (Figure 1). However, the introduced E or F chromosomes appeared to significantly increase seed protein and gluten contents. Therefore, the induction of wheat-*Ae. caudata* translocations involving chromosomes E or F could find application in breeding programs which targeted high-yielding or superior-quality wheat. Especially, the superior-quality wheat cultivars with high protein and wet gluten contents might be a candidate food that can provide nutrients for people with malnutrition.

In the aspect of marker development for *Ae. caudata* chromosomes, Peil et al. (1997, 1998) developed RAPD and SSR markers which could be used to detect *Ae. caudata* chromatin in a wheat background. Friebe et al. (1992) established the cytogenetic markers, namely the standard C-banding pattern of *Ae. caudata* chromosomes which could be used for *Ae. caudata* chromosome identification in wheat. Kong et al. (1999a,b) cloned specific repetitive DNA from the *Ae. caudata* genome and used it as a marker for the detection of *Ae. caudata* chromatin in wheat. Badaeva et al. (1996) described the pSc119 and pAs1 FISH karyotype together with the C-banding karyotype of the diploid *Aegilops* species including *Ae. caudata*. In this research, a total of 55 EST-based molecular markers which are specific for *Ae. caudata* chromosomes were developed (Table 3), providing new detection approaches for the quick selection and identification of wheat-*Ae. caudata* introgressions.

In the aspect of primer pair selection for suitable markers, the data of the current research showed that the rate of successful allocation of polymorphic EST-STS, EST-SSR, COS, and PLUG primers to *Ae. caudata* chromosomes was 3.6, 5.0, 3.7, and 12.4%, respectively. By comparison, the percentage for marker development of *Ae. markgrafii* and *Ae. cylindrica* using COS primers was 80.0% (Molnár et al., 2015), that for *Ae. umbellulata, Ae. comosa, Ae. speltoides*, and *Ae. markgrafii* using COS primers range from 46.49 to 53.38% (Molnár et al., 2016), that for *Ae. searsii* chromosomes by using EST-STS, EST-SSR, COS and PLUG primer was 1.0, 8.6, 5.7, and 16.7%, respectively (Gong et al., 2016), that for *Ae. mutica* chromosomes by using EST-STS, EST-SSR and PLUG primers was 2.0, 0, and 6.9%, respectively (Liu et al., 2015), that for *Ae. uniaristata* chromosomes by using EST-STS, EST-SSR, and PLUG primers was 3.5, 11.0, and 11.8%, respectively (Gong et al., 2014), that for *Ae. biuncialis, Ae. umbellulata, Ae. comosa, Ae. Biuncialis*, and *Ae. geniculata*, chromosomes rang) range from 54.1 to 80.3% by using COS primers (Molnár et al., 2013), that for *Ae. peregrina* with US chromosomes and the synthetic KU37 with US^sh^ chromosomes by using COS marker was 43.09%, respectively (Howard et al., 2011), and that for *Ae. ventricosa* chromsome was 27% by using COS primers (Burt and Nicholson, 2011). The percentage for marker development of *Lophopyrum elongatum* chromosomes by using EST-SSR and PLUG primers was 6.6 and 11.0%, respectively (Hu et al., 2012), and that for *Dasypyrum breviaristatum* chromosomes by using EST-STS and PLUG primer was 4.8 and 10.7%, respectively (Liu et al., 2011). The percentage for marker development by using different primer pairs varies. In this research, COS marker development rate using agarose gel electrophoresis is too much lower (3.7%) than that of other reports (27–80.3%) (Burt and Nicholson, 2011; Molnár et al., 2013, 2015, 2016), this might due to that the detection sensitiveness of capillary sequencer or silver staining is too much higher than agarose gel electrophoresis. In this research, PLUG primers appear to generate a higher percentage than other primer pairs, therefore, it should be the system of first choice for marker development of chromosomes belonging to Triticeae species when agarose gel electrophoresis was used. However, COS primer should also be a good choice for marker development if capillary sequencer or silver staining was used.

Schubert and Bluthner (1995) developed the set of wheat-*Ae. caudata* chromosome additions A–G. Among them, addition A was identified as chromosome 1C by use of isozymes, and also by chromosome characteristics such as the presence of a satellite and C-banding pattern. Friebe et al. (1992) identified additions B–G using the established standard C-banding pattern of the *Ae. caudata* chromosomes, assuming that additions C, D, and F might be 5C, 6C, and 3C. However, no molecular marker data at the time existed to support this conjecture. The marker results of our current research showed that 7, 19, and 11 markers could be used to identify additions C, D, and F, and among these markers, 6 (85.7%), 10 (52.6%), and 9 (81.8%) belong to homoeologous groups 5, 6, and 3 (Table 3). Therefore, additions C, D, and F should be chromosomes 5C, 6C, and 3C, which confirms Friebe's conjecture (Friebe et al., 1992). Furthermore, the recent single gene FISH mapping data for identifying homoeologous relationships of *Ae. caudata* chromosomes (Danilova et al., 2016, 2017) also supports this conclusion. Only six markers in this present study could be used to identify addition E, among them, three, two, and one marker(s) belong to homoeologous groups 7, 1, and 2. Addition line A was earlier shown to be the chromosome 1C addition (Schubert and Bluthner, 1995), therefore, addition E could not be addition 1C. The solitary homoeologous group 2 marker associated with addition line E was located on a chromosome terminal region (Table 3), while the two homoeologous group 7 markers were located on the subtelomeric regions of both chromosome long and short arm. Therefore, it is more likely that addition E might be the 7C addition, supporting the results of Danilova et al. (2016, 2017). Cytogenetic evidence has shown that the chromosome B of *Ae. caudata* might have relationship to both 4C and 5C chromosomes, while chromosome G of *Ae. caudata* might have a relationship to both 4C and 3C chromosomes (Friebe et al., 1992). Molecular data from this current research suggests that chromosome B of *Ae. caudata* not only has a relationship to homoeologous group 5, but also to groups 1, 2, and 3 (Table 3; Figure 4), however, we have not found a homoeologous group 4 marker herein. Meanwhile, chromosome G of *Ae. caudata* not only has a relationship to Triticeae groups 4 and 3, but also to groups 1 and 2 (Table 3; Figure 4). Seed high molecular weight (HWM) protein subunit evidence also supports the conclusion that addition G has a relationship to homoeologous group 1 (Han et al., 2015). Hence, complex chromosomal structural rearrangements might have occurred on chromosomes B and G of *Ae. caudata*. The molecular data of this research, the recent molecular evidence (Molnár et al., 2016) and cytogenetic evidence (Danilova et al., 2016, 2017) all support that chromosomal structural rearrangements have occurred on chromosomes of *Ae. caudata*, therefore, further studies into the detailed structures of each of these *Ae. caudata* chromosomes are warranted.

Wheat-alien introgressions play an important role in wheat resistance breeding. The most notable examples are the wheat-rye 1BL.1RS translocation (Rabinovich, 1998) which for many years was part of most wheat cultivars grown around the world, and also wheat-*Dasypyrum villosum* 6VS/6AL (Cao et al., 2011) translocation carrying the powdery mildew resistance gene *Pm21*. Therefore, production of novel wheat-alien species translocations, particularly for disease resistance, has always been the research hot topic. Non-compensating translocations are rarely used in wheat breeding due to the genetic drag or bad agronomical traits (Sears, 1993; Friebe et al., 1996). Therefore, identification of whether the target wheat and alien species chromosomes have been structurally rearranged or not is essential before embarking on the exhaustive and time consuming task of trying to produce compensating translocations for commercial agriculture. Based on the molecular data of this research (Table 3), we found evidence that chromosomes of *Ae. caudata* have been structurally rearranged. However, evidence from standard C-banding patterns of *Ae. caudata* chromosomes suggested that no chromosomal arrangements had occurred (Friebe et al., 1992), but in contrast, single gene FISH data support the conclusion that *Ae. caudata* chromosomes had undergone extensive structural rearrangments (Danilova et al., 2016). Moreover, our molecular data indicate that the rearrangements of B–G chromosomes of *Ae. caudata* involved at least two homoeologous groups, therefore, a large population and intensive screening pressure needs to be taken into consideration when inducing and searching for wheat-*Ae. caudata* compensating translocations.

## Author contributions

CL and JL conceived and designed the experiments. RH, HL, JS, and GL performed the experiments. AL and XC contributed reagents/materials/analysis tools. HY, CL, and JG performed disease resistance testing. SZ, DC, and ZZ analyzed the data. WG and RH wrote the paper.

### Conflict of interest statement

The authors declare that the research was conducted in the absence of any commercial or financial relationships that could be construed as a potential conflict of interest.
